# Escitalopram oxalate-loaded chitosan nanoparticle *in situ* gel formulation intended for direct nose-to-brain delivery: *in vitro*, *ex vivo*, and *in vivo* pharmacokinetic evaluation

**DOI:** 10.3389/fphar.2025.1577331

**Published:** 2025-03-28

**Authors:** Md Ali Mujtaba, Md Abdur Rashid, Mangesh D. Godbole, Yahya Alhamhoom, Divyashri S. Shende, Sameer Alshehri, Mohammad J. Akbar, Mohammed Kaleem, Ujwala N. Mahajan, Naiyer Shahzad

**Affiliations:** ^1^ Department of Pharmaceutics, Faculty of Pharmacy, Northern Border University, Arar, Saudi Arabia; ^2^ Center for Health Research, Northern Border University, Arar, Saudi Arabia; ^3^ Department of Pharmaceutics, College of Pharmacy, King Khalid University, Abha, Saudi Arabia; ^4^ Department of Pharmaceutical Analysis, Dadasaheb Balpande College of Pharmacy, Rashtrasant Tukadoji Maharaj Nagpur University, Nagpur, Maharashtra, India; ^5^ Department of Pharmaceutics and Industrial Pharmacy, College of Pharmacy, Taif University, Taif, Saudi Arabia; ^6^ Department of Pharmaceutics, College of Clinical Pharmacy, Imam Abdulrahman Bin Faisal University, Dammam, Saudi Arabia; ^7^ Department of Pharmacology, Dadasaheb Balpande College of Pharmacy, Rashtrasant Tukadoji Maharaj Nagpur University, Nagpur, Maharashtra, India; ^8^ Department of Pharmacology and Toxicology, Faculty of Medicine, Umm Al-Qura University, Makkah, Saudi Arabia

**Keywords:** intranasal drug delivery, escitalopram, chitosan, nanoparticles, *in situ* gel, pharmacokinetics

## Abstract

**Introduction:** Escitalopram oxalate (ESCI) is a biopharmaceutical classification system (BCS) class II antidepressant drug, that suffers limited oral bioavailability due to extensive hepatic metabolism. Therefore, this study aimed to develop and evaluate chitosan nanoparticles (CSNPs) embedded in an *in situ* gel for intranasal (i.n) drug delivery.

**Methods:** ESCl-loaded CSNPs were prepared by the ionic gelation method and were optimized using 3^2^ factorial design. The optimized CSNPs were incorporated into pH-sensitive *in situ* gel composed of carbopol 940 and HPMC K4M for i. n administration.

**Results:** The optimized CSNPs exhibited a particle size of 189 ± 3.14 nm, polydispersity index 0.372 ± 0.84, zeta potential 22.2 ± 1.25 mV, and entrapment efficiency of 76.5% ± 1.64%. FTIR, DSC, and XRD analysis of CSNPs confirmed the encapsulation of the ESCI within the formulation. The *in vitro* drug release profile of the ESCI-loaded CSNPs *in situ* gel exhibited an initial burst release followed by a slow and sustained release phase. The *in situ* gel studies demonstrated that 80.72% ± 3.12% of the drug permeated within 8 h through the goat nasal mucosa in *ex vivo* permeation studies. In pharmacokinetic studies, the C_max_ in the brain following a single nasal administration of ESCI-loaded CSNPs *in situ* gel was 4.67 folds higher than the oral solution. The total AUC_0-12_ *in situ* gel was 3.40 times higher than the i. n drug solution and 13.31 times higher than an oral solution. The mean residence time (MRT) for the brain’s CSNPs *in situ* gel was higher than i. n drug and oral solutions.

**Conclusion:** This higher C_max_ and prolonged MRT in the brain highlight the potential of CSNPs *in situ* gel as an effective brain-targeting system via the intranasal route. These results indicate that i. n delivery of the ESCl-loaded CSNPs *in situ* gel is a promising strategy for controlled release of ESCI, enhancing therapeutic efficacy and mitigating the disadvantages of oral delivery.

## 1 Introduction

Depression is one of the most prevalent central nervous system (CNS) disorders worldwide, characterized by symptoms such as persistently low mood, fatigue, sleep disturbances, loss of appetite, reduced interest in activities, feelings of guilt or low self-worth, poor concentration, and anxiety (Cui et al., 2024; More et al., 2025a). These symptoms may become chronic, significantly affect daily life functions, and in severe cases may lead to suicide. Worldwide, depression affects about 350 million individuals annually, and the incidence of suicides is rising every year, according to the World Health Organization (WHO) (Agrawal et al., 2018). Selective serotonin reuptake inhibitors (SSRIs) are the preferred treatment due to their ease of administration and relatively mild side effects. Escitalopram oxalate (ESCI), SSRI, is commonly used for managing major depressive disorder (MDD) and general anxiety disorder (GAD), and is approved by the FDA as an antidepressant, that is typically administered orally for these conditions (Jin et al., 2022). However, achieving adequate drug concentrations in the brain is crucial for effectively treating CNS disorders. Approximately 56% of ESCI binds to human plasma proteins and is primarily metabolized in the liver, resulting in the formation of less lipophilic compounds: dimethyl-escitalopram and dimethyl-escitalopram, which leads to limitations in crossing the brain barrier. Further, ESCI belongs to the category of BCS class II drugs, which have low water solubility. Its decreased systemic and cerebral bioavailability is partially due to the combined effects of these two factors. Along with this, delayed delivery to the brain and peripheral side effects of ESCI, trigger the need for an alternative route that directly delivers the drug to the brain.

The intranasal (i.n) route is a non-invasive and effective strategy for delivering drugs directly from the nasal mucosa to the brain, offering a rapid method to treat central nervous system disorders with minimal exposure to systemic circulation (Rabiee et al., 2021). A rich vascular network and high permeability of the nasal mucosa enable quick absorption and prompt therapeutic effects ^(2)^. Recently, intranasal delivery has gained popularity for a range of applications for topical, systemic, and targeted brain treatments. This route bypasses the challenges of extensive hepatic first-pass metabolism, and stringent acidic conditions inside the gastrointestinal tract, providing a better pharmacokinetic profile for the lipophilic drugs and facilitating brain targeting via the olfactory pathway (Md et al., 2018). Because of their non-invasive nature, intranasal formulations are also more convenient for patients (Kaur et al., 2015; Modgill et al., 2016). In this context, chitosan (CS) has become a widely researched material for its utility in nanostructured intranasal drug delivery systems. CS, a natural polysaccharide derived from chitin, possesses unique physicochemical properties, including biocompatibility, biodegradability, and nontoxicity. These characteristics render it a suitable candidate for nanoparticle formulation, particularly in the context of intranasal delivery (Uppuluri et al., 2021). Furthermore, because of its intrinsic positive charge at physiological pH, CS has mucoadhesive qualities that enable it to adhere to negatively charged mucosal surfaces, extending the residence time and improving absorption of the encapsulated drugs. CS differs from conventional polymeric NPs in several ways, which makes it very useful for biological applications and targeted drug administration (Omidian et al., 2024). Chitosan nanoparticles (CSNPs) have been demonstrated to facilitate medication transport across the blood-brain barrier, improving bioavailability, enhancing brain delivery, and reducing systemic side effects. These properties position CS as a promising excipient for developing efficient, scalable, and patient-friendly i. n drug delivery systems with potential applications in neurological and systemic drug therapies (Ahmad et al., 2016; Omidian et al., 2024).

Over the past 3 decades, there has been an increasing emphasis on the advancement of controlled and sustained drug delivery systems. Considerable research efforts have been directed toward developing polymer-based systems, such as *in situ* gels (Kaur et al., 2015). These systems have garnered significant interest recently. They begin as liquid aqueous solutions and form gels under physiological conditions. The ability to provide consistent, prolonged drug release, combined with biocompatibility, stability, and reliable medication levels, enhances their precision (Malik et al., 2015; Modgill et al., 2016). Among the CS-based nanocomposites, nanoparticles (NPs) formed through ionotropic gelation are the most extensively studied. The process involves the sol-gel transition of CS polymers induced by their interaction with a poly-anionic crosslinking agent, commonly sodium tripolyphosphate (STPP) (Calvo et al., 1997; Sreekumar et al., 2018)^.^ Amongst numerous NPs prepared from biodegradable polymers, CS-based NPs are among the most promising delivery systems for cerebral disease therapy and diagnosis because of their unique characteristics. CS have primary amine groups of the glucosamine residues at the C-2 position, adding vital functional properties to it that can be used for bio-fabrication into nanoparticles (Prabaharan, 2015). Research has highlighted that CS exhibits unique mucoadhesive properties, allowing it to adhere to the nasal mucosa for extended durations, enhancing drug bioavailability. This adhesion slows mucociliary clearance, enabling prolonged contact time for the drug. Additionally, the cationic nature of CS enhances paracellular transport by temporarily opening tight junctions between epithelial cells. This mechanism increases the permeability of the nasal epithelium, thereby improving drug absorption efficiency (Omidian et al., 2024). Carbopol is a pH-sensitive polymer used for gel preparation at nasal pH, which seems to improve drug release and direct nose-to-brain drug delivery.

This finding aims to formulate and evaluate ESCl-loaded chitosan nanoparticles (CSNPs) in an *in situ* gel, intended for treating depression via a non-invasive intranasal route, facilitating direct drug delivery from nose to brain. The formulation process involves optimizing the CS-to-STPP ratio to achieve efficient drug loading, using a 3^2^-factorial design (two factors, three levels). Following optimization, the ESCl-loaded CSNPs were incorporated into a pH-sensitive carbopol gel to develop an ESCl-loaded CSNP *in situ* gel, which underwent comprehensive physicochemical characterization. The study also investigates the *in vitro* drug release, *ex vivo* permeation, and comparative pharmacokinetics of the ESCl-loaded CSNP *in situ* gel against an ESCl solution to assess brain and blood distribution profiles.

## 2 Material and methods

### 2.1 Materials

CS with a molecular weight of ∼750,000 Da and 85% deacetylation, along with STPP, was obtained from Sigma-Aldrich, Bangalore, India. ESCI was provided as a gift sample by Ajanta Pharmaceuticals Ltd., Mumbai, India. Glacial acetic acid, carpool, potassium dihydrogen phosphate, and sodium hydroxide were sourced from Loba Chemie Pvt. Ltd., Mumbai, India, while HPMC K4M was supplied by Colorcon Asia Pvt. Ltd., Goa, India.

### 2.2 Methods

#### 2.2.1 Preparation of chitosan nanoparticles (CSNPs)

CSNPs were synthesized using the ionic gelation technique (Alam et al., 2012). This involves gradually adding an STPP solution (2 mg/mL) into a CS solution (1 mg/mL), prepared in 1% glacial acetic acid, with continuous stirring at room temperature. CSNPs were formed because of ionic interactions between the positively charged amino groups of CS and the negatively charged groups of STPP. Based on initial findings, the CS/TPP ratio was identified (Table 1). For ESCI-loaded CSNPs, the same procedure was followed, maintaining the CS/TPP ratio, while varying the amount of ESCI added to the CS solution before TPP addition. The resulting NPs were concentrated at 10,000 × g at 4 C for 30 min. The supernatant was analyzed to evaluate particle size (PS), zeta potential (ZP), drug-loading (DL) capacity, and encapsulation efficiency (EE), while the pellets were cleaned with distilled water and lyophilized for further characterization studies (Rabiee et al., 2021).

**TABLE 1:** Preliminary batches of nanoparticles.

Formulation code	Concentration of CS (mg/mL)	Concentration of TPP (mg/mL)	PS (nm)	PDI	%EE
NP-1	0.5	1	264 ± 3.81	0.35 ± 0.018	72.22 ± 4.83
NP-2	0.5	1.5	230 ± 2.96	0.26 ± 0.011	75.44 ± 3.65
NP-3	0.5	2	229 ± 3.18	0.28 ± 0.014	80.0 ± 4.16
NP-4	1	1	208 ± 3.55	0.22 ± 0.019	70.32 ± 2.96
NP-5	1	1.5	202 ± 2.38	0.36 ± 0.018	74.24 ± 3.56
NP-6	1	2	189 ± 2.92	0.37 ± 0.021	76.5 ± 2.45
NP-7	1.5	1	229 ± 3.63	0.33 ± 0.024	65.4 ± 4.58
NP-8	1.5	1.5	202 ± 4.18	0.29 ± 0.181	67.71 ± 4.04
NP-9	1.5	2	164 ± 2.87	0.22 ± 3.8	73.34 ± 3.73

#### 2.2.2 Formulation optimization using the design of experiment (DoE)

A 3^2^ (two-factor and three-level) factorial design was used for optimizing ESCI-loaded CSNPs. Three concentration levels (low, middle, and high) of CS and STPP were selected as independent variables. The dependent variables were chosen as: particle size (PS), zeta potential (ZP), and entrapment efficiency (%EE) (Alam et al., 2012). A 3^2^ factorial design was used in the Design Expert^®^ (Version 13 Stat-Ease, Inc. Minneapolis, MN) to formulate 12 formulations (Valderrama N et al., 2020). After undergoing multiple regression analysis on the modified values of the independent variables in the factorial design, a full-model polynomial equation was derived. Every run was subjected to an ANOVA, and integrated software tools were used for statistical validation. The results indicated that quadratic, 2FI, cubic, and linear models were appropriate for the statistical analysis of coefficient effects. Three-dimensional plots were generated using Design Expert^®^ software to illustrate the impact of the variables, and multiple checkpoints were assessed to confirm response characteristics. Prediction accuracy was evaluated by comparing data-derived and observed outcomes.

#### 2.2.3 Characterization of CSNPs

##### 2.2.3.1 PS, PDI and ZP measurement

Following appropriate dilutions with distilled water, hydrodynamic PS and ZP were determined by photon correlation spectroscopy (PCS) (Zetasizer Nano-ZS-90, Malvern Instruments, Worcestershire, United Kingdom). The consistency of nanoparticle sizes and the size distribution within the sample were assessed using the polydispersity index (PDI). Formulation stability and interactions with biological membranes are significantly influenced by the zeta potential, which represents the surface charges of the nanoparticles (Gao et al., 2022; Singh et al., 2015). The preparation of ZP measurement samples was equivalent to that of PS measurement samples. PCS also measured ZP for three replicates of each sample.

##### 2.2.3.2 EE and DL measurement

EE and DL were measured using the indirect method (Dalvi et al., 2021; Haque et al., 2014). NPs were separated from the aqueous medium containing unbound ESCI by centrifugation at 15,000 rpm for 45 min at 4°C to calculate the %EE and %DL of the NPs. The amount of free ESCI in the supernatant was measured using a UV spectrophotometer at 237 nm. All measurements were made in triplicate, and the % EE and %DL of ESCI-loaded CSNPs were calculated using the formula below.

$$\%\text{EE}=\frac{\text{Total}\;\text{drug}\;\text{added}\;–\;\text{Unentrapped}\;\text{drug}}{\text{Total}\;\text{drug}\;\text{added}}\;\times \;100$$

$$\%\text{DL}=\frac{\text{Total}\;\text{drug}\;\text{added}\;–\;\text{Unentrapped}\;\text{drug}}{\text{weight}\;\text{of}\;\text{nanoparticles}}\;\times \;100$$

##### 2.2.3.3 Fourier-transform infrared (FTIR) spectroscopy analysis

FTIR spectroscopy (Shimadzu 8400S, Japan) of the drug and excipients (ESCI, CS, STPP, Carbopol 940, HPMC K4M, and optimized NPs formulation) were performed using the potassium bromide pellet technique. After mixing 1–3 mg of the material with dry potassium bromide, it was examined in transmission mode throughout a wave number range of 4,000–400 cm^−1^ (Fadlelmoula et al., 2022; Kharwade et al., 2024).

##### 2.2.3.4 Differential scanning calorimetry (DSC) analysis

DSC analysis for the pure drug, excipients, and optimized NPs formulation was performed using a DSC instrument (DSC-1821e, Mettler- ToledoAG, Analytical, Schwerzenbach, Switzerland). 5 mg samples were put into aluminum pans and sealed. The probes were exposed to extreme heat in the presence of nitrogen from 50 to 400°C at a rate of 10°C per minute. DSC is an effective tool for examining the thermal behavior of many kinds of materials. Evaluating the melting, deterioration, compatibility, stability, and numerous other associated characteristics of test materials may produce significant data by determining the alterations in material characteristics as a result of controlled temperature variations (Gill et al., 2010).

##### 2.2.3.5 X-ray diffraction study (XRD) analysis

XRD analysis was performed on the pure drug and a physical mixture of the drug with excipients, and the optimized NP formulation to evaluate the impact of qualitative and quantitative factors on crystallinity. The experiment was performed using a D2 Phaser 2^nd^ generation XRD apparatus (Bruker AXS, Inc., Madison, WI, United States). Data were recorded at a voltage of 30 kV, 10 mA current, and a 2θ angle range of 10°–80°. The scanning was performed at a rate of 50 min across diffraction angles of 50–400 (2θ) (Fadlelmoula et al., 2022; Zhang et al., 2014).

#### 2.2.4 Preparation of *in situ* gel from ESCl-loaded CSNPs

Nasal *in situ* gel of ESCl was prepared using the cold method (Thakkar and Prajapati, 2021). The cold method involved the slow addition of polymers (Carbopol 940 and HPMC K4M), lyophilized CSNPs containing 5 mg of ESCl, and additives in cold water with continuous agitation. The formed mixture was stored overnight at 4°C to get a clear gel. 1% benzalkonium chloride was used as a preservative to increase the shelf-life of the product. The solution was kept in the refrigerator for subsequent usage (Naresh et al., 2020).

#### 2.2.5 Evaluation of *in situ* gel formulation for appearance/clarity, pH, viscosity, gel strength, and drug content determination

The nasal formulation was observed for color and the presence of suspended particulate matter, if any. The clarity of the solution was further assessed by observing them against a dark and white background (Nirmal et al., 2010). The pH of the formulation was determined using a pH meter that was calibrated using buffers of pH 7 and pH 9 before the measurements (Nandgude et al., 1970). The measurements were taken three times, and the results were given as mean ± SD. The viscosity of the formulation was measured using a Brookfield viscometer at 10 rpm. A narrow body beaker containing about 10 mL of the formulation was put suitably under the spindle (S-92) of the viscometer (Qi et al., 2021). The gel strength was tested by placing a 20 g standard weight on 50 g of gel. The time required to penetrate 5 cm deep into the cylinder was measured in seconds (Godbole et al., 2014). The drug concentration was evaluated by diluting 1 mL of the formulation with 100 mL of distilled water and shaking vigorously. 10 mL of this solution was removed and diluted with distilled water to make 100 mL. The solution’s absorbance was measured at 237 nm with a UV-Vis spectrophotometer, and the quantity of ESCl in the sample was estimated using a calibration curve.

#### 2.2.6 *In vitro* drug release study

The *in vitro* drug release study was conducted by using dialysis bag diffusion techniques (Godbole et al., 2014; Yasir and Sara, 2014). The *in vitro* release rates of ESCI-loaded gel and optimized ESCI-loaded CSNPs *in situ* gel were performed in a dissolving medium containing simulated nasal fluid (SNF) at pH 6.4. The *in situ* gel formulation (5 mg of drug) was filled into a pre-moistened cellulose-acetate dialysis bag (2.0 mL) and sealed at both ends. The receptor compartment was filled with phosphate buffer (pH 6.4) and maintained at 37°C ± 0.2°C. Because of the system’s pH, the solution quickly formed a gel and remained attached. To keep the sink condition, 1 mL of the solution from the reservoir compartment was taken at 1, 2, 3, 4, 5, 6, 7, and 24 h and replaced with a fresh buffer. After a suitable dilution, the samples were applied with the UV spectrophotometer at 237 nm.

#### 2.2.7 *Ex vivo* drug permeation study

The *ex vivo* drug permeation study was conducted by using goat nasal mucosa (Godbole et al., 2014). Double-distilled water was used for washing newly sliced nasal mucosa and was obtained from a slaughterhouse. After washing, it was immediately placed in a standard saline solution. A piece of approximately 2 cm^2^ was cut and suitably inserted between the donor and acceptor compartments. The acceptor compartment was filled with pH 6.4 phosphate buffer and maintained at 37°C ± 0.2°C. 1 mL of *in situ* gel containing 5 mg of the drug was applied to the mucosa that was placed at the donor compartment. Because of the system’s temperature, the solution immediately formed a gel and remained adhered to it. At predefined time intervals (0.25, 1, 2, 4, 6, 8, 10, 12, and 24 h), 1 mL of the reservoir compartment’s solution was taken and replaced with fresh buffer. Following adequate dilution, the samples were analyzed with a UV spectrophotometer at 237 nm.

#### 2.2.8 *In vivo* pharmacokinetics and tissue distribution study

The Institutional Animal Ethical Committee at Dadasaheb Balpande College of Pharmacy, Nagpur, India, reviewed and approved this work, with the protocol number DBCOP/IEAC/1426/2022-23/P-16. This investigation was carried out on male Sprague Dawley rats weighing 200–250 g. Rats were placed into three groups of six each. Group 1 received an intranasal dose of ESCI-loaded CSNPs *in situ* gel solution equivalent to 0.101 mg. Group II was given an oral ESCI suspension dose of 1.01 mg. Group III was given an intranasal solution of ESCl containing 0.101 mg. After dosing, the animals were housed/kept aside and at predetermined time intervals, the animals were euthanized by using diethyl ether followed by cervical dislocation. After sacrifice, blood was collected in an EDTA tube, and the brain was carefully isolated and cleaned properly with double distilled water. The brain was weighed, and acetonitrile (HPLC grade) was added 3 times the brain’s weight. The brain homogenate was prepared and centrifuged for 15 min at 10,000 rpm. Around 100 μL of the supernatant was withdrawn carefully and dried with nitrogen gas in a test tube. 1 mL of 1 µg internal standard was added in the Eppendorf tube and then the sample was analyzed using HPLC. For the blood sample, the collected amount of blood was directly centrifuged at 10,000 RPM for 15 min. Then 100 μL plasma supernatant was withdrawn and 1 mL of HPLC grade acetonitrile was added to precipitate the plasma proteins. Then, this solution was centrifuged at 10,000 rpm for 15 min. After centrifugation, the supernatant was withdrawn carefully and dried with nitrogen gas in a borosilicate glass test tube. 1 mL of 1 µg internal standard was added to the eppendorf tube. The sample was analyzed using HPLC (Silva et al., 2021). The ESCI quantification calibration curve was obtained between a concentration range of 5 ng/mL to 10 μg/mL with a high co-efficient of correlation (*R* ^2^ = 0.999). The pharmacokinetics of ESCI were determined post-dosing using pharmacokinetic software (PK Functions for Microsoft Excel, Pharsight Corporation, Mountain View, CA) from the plasma concentration-time profile of ESCI.

#### 2.2.9 Statistical analysis

All findings are expressed as mean ± SD, which was determined using data from three independent studies. A t-test was used to examine all experimental data, and a p-value <0.05 indicated significant differences.

## 3 Results and discussion

### 3.1 Formulation and evaluation of ESCI-loaded CSNPs

ESCI-loaded CSNPs were prepared by ionic gelation method. Various CS and TPP concentrations were tested to determine the optimal CS/TPP ratio based on PS, PDI, and %EE. Table 1 depicts the mean PS, PDI, and %EE for the various batches of CS-NPs. The ionic reaction among the negatively charged TPP groups and the positively charged amino groups of CS led to the production of NPs. Based on the preliminary research, the CS/TPP ratio was determined. The same technique was used for producing ESCI-loaded CSNPs, and the CS/TPP ratio remains constant. NPs were collected by lyophilization (Alam et al., 2012).

### 3.2 Experimental design and data analysis

The ESCI-loaded CSNPs were optimized using a 3^2^ (two-factor and three-level) factorial design. CS and STPP were chosen as independent variables that were varied at three levels (low, middle, and high) (Mujtaba et al., 2024a). PS, ZP, and %EE were selected as dependent variables (Table 2). The trial runs of twelve batches was provided by Design-expert software version 13 (Stat-Ease, Inc., Minneapolis, MN), which is shown in Table 3. Determination of the experimental components affecting the NPs’ physicochemical properties was made feasible by the applied technique. The statistical regression models were calibrated independently for each of the dependent variables. Furthermore, the statistical significance of the coefficients, *R2* values, and normal distribution of the residues was used for the statistical validation of the polynomial equations via an ANOVA (Analysis of Variance) technique (Valderrama N et al., 2020).

**TABLE 2:** Variables used in 3^2^ factorial designs.

Independent variables	Levels			Dependent variables
	Low (−1)	Medium (0)	High (+1)	
Concentration of CS (mg/mL)	0.5	1	1.5	PS (nm) ZP (mv) %EE
Concentration of STPP (mg/mL)	1	1.5	2	

**TABLE 3:** Results of the 3^2^ factorial design variables.

Batch no.	Independent variables		Dependent variables		
	Factor 1 Concentration of CS (mg/mL)	Factor 2 Concentration of STPP (mg/mL)	Response 1 PS (nm)	Response 2 ZP (mv)	Response 3 %EE
1	0.5	1	264	28.5	72.22
2	1	1	208	22.5	70.32
3	1.5	1	229	24.3	65.4
4	0.5	1.5	230	27.3	75.44
5	1	1.5	202	21.1	74.24
6	1.5	1.5	201	20.8	67.71
7	0.5	2	229	19.5	80.0
8	1	2	189	22.2	76.5
9	1.5	2	164	20.5	73.34
10	1	1.5	202	21.1	74.24
11	1	1.5	202	21.1	74.24
12	1	1.5	202	21.1	74.24

#### 3.2.1 Response 1: effect on PS

PS of all batches was in the range of 164–230 nm and PDI was found to be in the range of 0.22–0.36 which is in the acceptable range. The model that indicates the effect of factors on the PS is illustrated in the following equation:

$$\text{PS}=201.71\;–\;15.83A\;–\;14.33B\;–\;15.75\text{AB}+13.88A^{2}\;–\;2.62B^{2}$$

**FIGURE 1:**
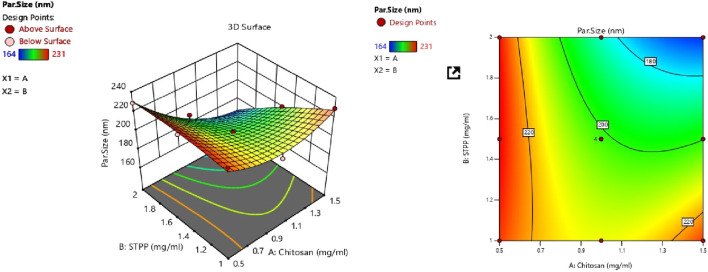
Contour plot and 3D surface response plot showing the effect of CS vs. STPP on PS.

The equation expressed in coding makes it easier to estimate the response which is associated with a specific quantity of every factor. By comparing the coefficients of the various elements, this coded formula is useful for assessing their relative significance. The two variables procedure (A and B) and their association have a quantitative effect on the given responses, as revealed by the polynomial equations. As shown in Table 4, a quadratic model for ZP was determined using ANOVA. Model terms that have *p*-values less than 0.0500 are considered significant. Significant model terms in this case include A, B, AB, and A^2^. The F-value of 62.10 indicates that the lack of fit is insignificant. CS and STPP have significant effects on PS. When the concentration of CS was increased, the PS decreased and after a concentration of 1 mg/mL, it was increased. This may be due to the aggregation of particles at higher concentrations of CS, which may lead to an increase in PS (Mujtaba and Alotaibi, 2023). The PS decreases as the STPP concentration increases until it reaches a threshold; however, a further increase in the STPP concentration results in particle aggregation. From Figure 1 it was concluded that CS and STPP have a significant effect on PS as the concentration of CS increases, the PS decreases and then increases. As the concentration of STPP increases, PS decreases. The predicted *R* ^2^ of 0.8055 lines up with the adjusted *R* ^2^ of 0.9652, with a difference of less than 0.2. The signal-to-noise ratio is determined by the level of precision. A ratio higher than 4 is recommended. The ratio of 24.595 indicates that the signal is adequate. This demonstrates that the model may be used to navigate the design space. Surface plots provide a graphical representation of the data, making it possible to visualize these interactions. The response surface curve in Figure 1 illustrates the effect of both CS and STPP concentrations on the PS.

**TABLE 4:** Results of ANOVA.

Response model	Sum of squares	Degree of freedom	Mean square	F Value	Model *p* value	*R* ^2^	Adeq. Precision
PS	4,254.46	5	850.89	62.10	0.0001	0.9819	24.59
ZP	44.38	2	22.19	24.19	0.0002	0.8438	14.37
% EE	162.15	2	81.07	72.68	0.0001	0.9417	27.84

#### 3.2.2 Response 2: effect on ZP

ZP of all batches ranged from 19.5 mV to 28.5 mV as shown in Table 3. The following polynomial equation describes the mathematical link between the formulation’s independent variables and ZP. The polynomial equation contributes to understanding the link between the variables and ZP.

ZP=21.56 – 0.8500A – 2.58B

**FIGURE 2:**
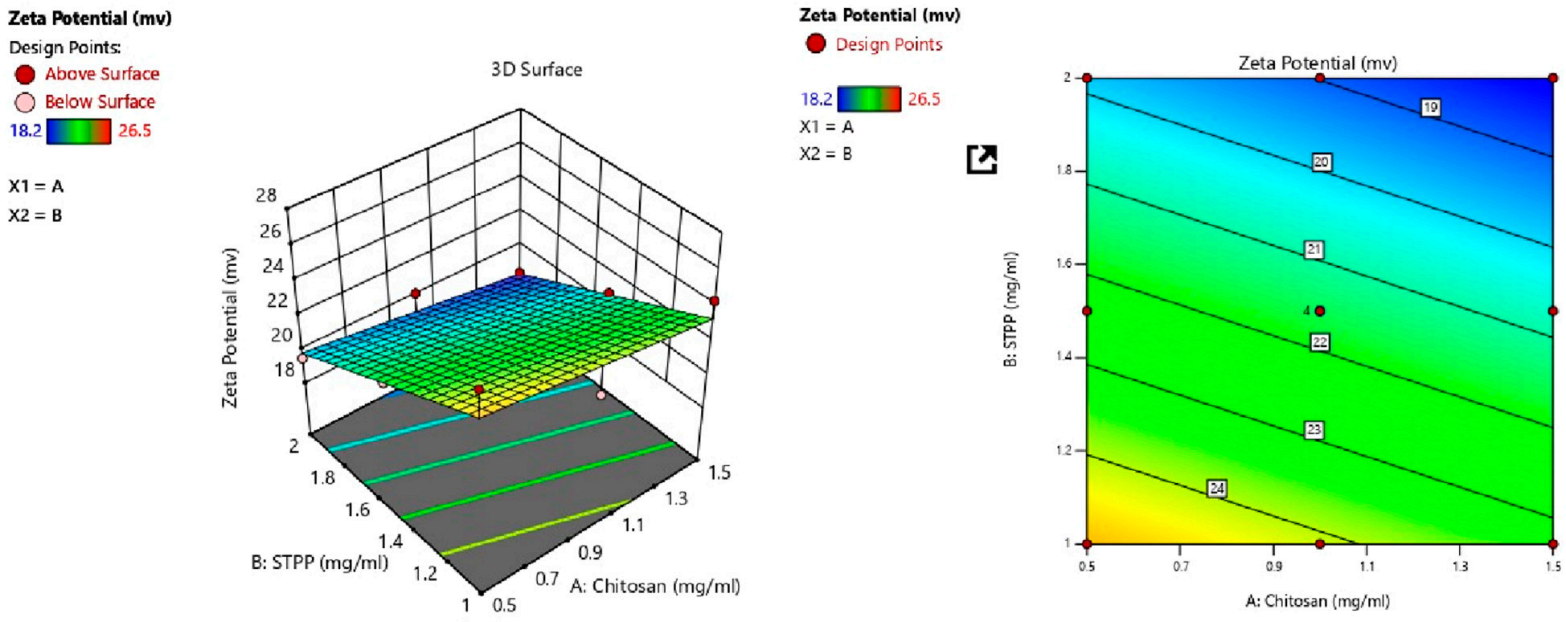
Contour plot and 3D surface response plot showing the effect of CS vs. STPP on ZP.

CS and STPP significantly affect ZP when the concentration of CS increases, ZP decreases, and when STPP is increased, ZP also decreases. ANOVA was used to develop the linear model for ZP, as shown in Table 4. The model’s F-value of 24.32 indicates that the model is significant. P-values <0.0500 imply that the model terms are significant. In this scenario, B is an important model term. Values above 0.0500 imply that the model terms are not significant. The F-value of 62.10 indicates that the lack of fit is insignificant. The predicted *R* ^2^ of 0.6566 is comparable to the adjusted *R* ^2^ of 0.8091, with a difference of less than 0.2. Adequate precision assesses signal-to-noise ratio. A ratio greater than four is preferred. A ratio of 14.377 shows an appropriate signal. This suggests that the model can be used to navigate the design space. From Figure 2 it was concluded that CS and STPP have significant effects on ZP as the concentration of CS increases ZP decreases and with the increase in STPP concentration, ZP decreases.

#### 3.2.3 Response 3: effect on % EE

The following equation can be used to determine the relative impact of the components by comparing the factor coefficients (Table 3).

%EE=73.24 – 3.70A+3.65B

**FIGURE 3:**
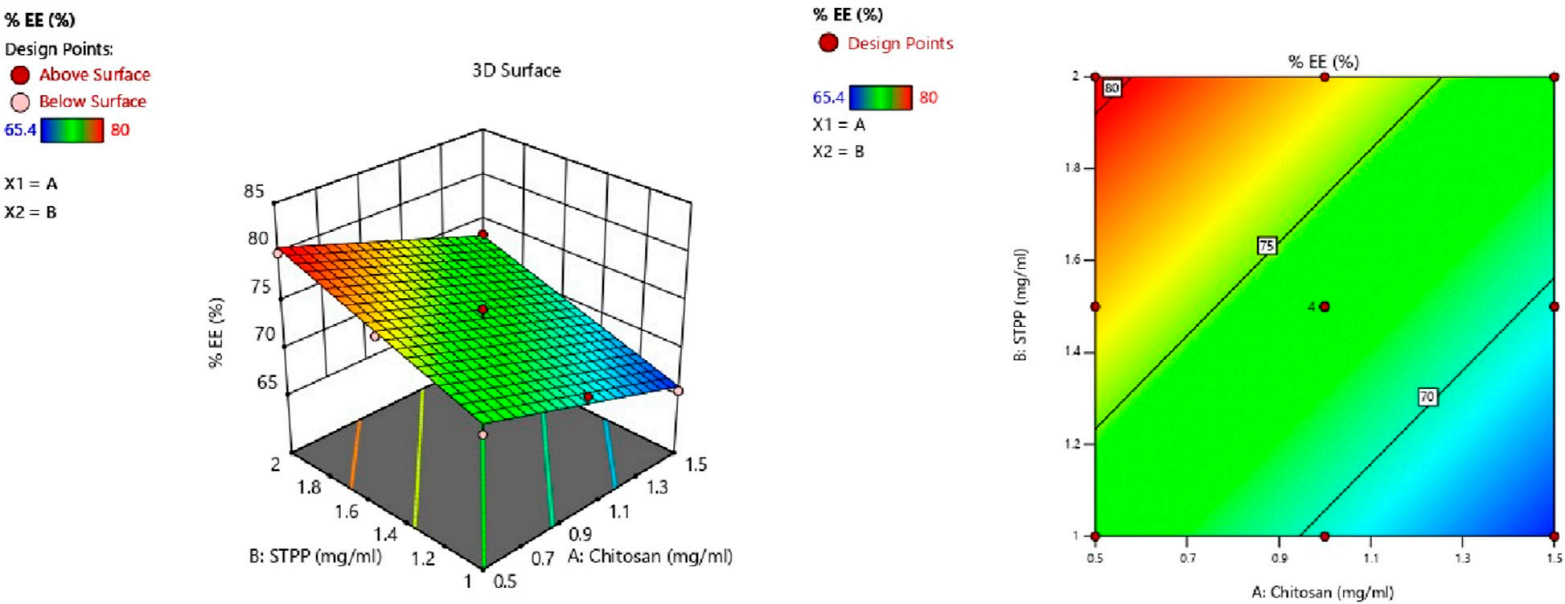
Contour plot and 3D surface response plot showing the effect of CS vs. STPP on %EE.

CS and STPP have significant effects on %EE. When the concentration of CS increased, the % EE decreased, whereas an increase in STPP concentration led to an increased % EE. The % EE of all the batches was in the range of 65.4%–80.0%. This trend may be attributed to the higher concentration of CS, which increases the viscosity and may hinder the effective crosslinking with STPP, which results in reduced drug entrapment. On the contrary, a higher amount of STPP reinforces ionic crosslinking, leading to enhanced drug entrapment. ANOVA was employed to establish the linear model for %EE, as shown in Table 4. The predicted *R* ^2^ values of 0.8981 closely align with the adjusted *R* ^2^ value of 0.9287, with a difference of less than 0.2. Adequate precision, which evaluates the signal-to-noise ratio, yielded a value of 27.843, exceeding the desired threshold of 4 and signifying a strong signal suitable for navigating the design space. The model F-value of 72.68 indicates that the lack of fit is insignificant. The non-significant lack-of-fit indicates that the pattern in the data can be adequately described by the model. The effect of the two variables is also evident in Figure 3, as it shows both CS and STPP concentrations affecting the %EE significantly, such that CS concentration negatively affects %EE whereas STPP concentration positively affects %EE.

### 3.3 Check-point analysis and validation of ESCI-loaded CSNP formulation

**FIGURE 4:**
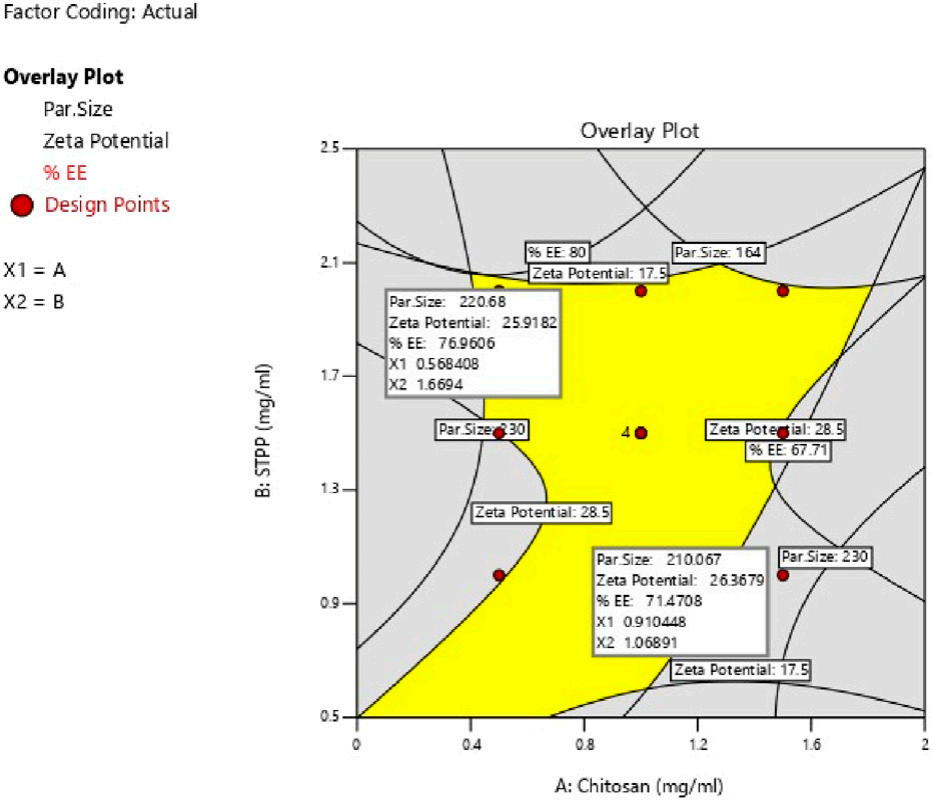
Overlay plot of response variables Batch A1 and A2.

The study determined the optimal values of independent variables by analyzing their effects on the responses. Overlay plot analysis was employed to identify checkpoint batches (Figure 4). Based on this study, two checkpoint batches, A1 and A2, were formulated and then analyzed for PS, ZP, and %EE, as shown in Table 5. When the actual values were compared to the predicted values, the difference was less than 5% of all responses.

**TABLE 5:** Predicted and actual response of checkpoint batch.

Evaluation parameters	Batch A1			Batch A2		
	Predicted value	Actual value	% Error	Predicted value	Actual value	% Error
PS	210.06	214.1 ± 2.57	1.89%	220.68	229.51 ± 1.24	3.84%
ZP	26.36	27.0 ± 1.56	2.37%	25.91	26.10 ± 3.25	0.72%
% EE	71.47	72.51 ± 1.252	1.43%	76.96	77.97 ± 1.254	1.29%

### 3.4 Optimization of ESCI-loaded CSNPs formulation

**FIGURE 5:**
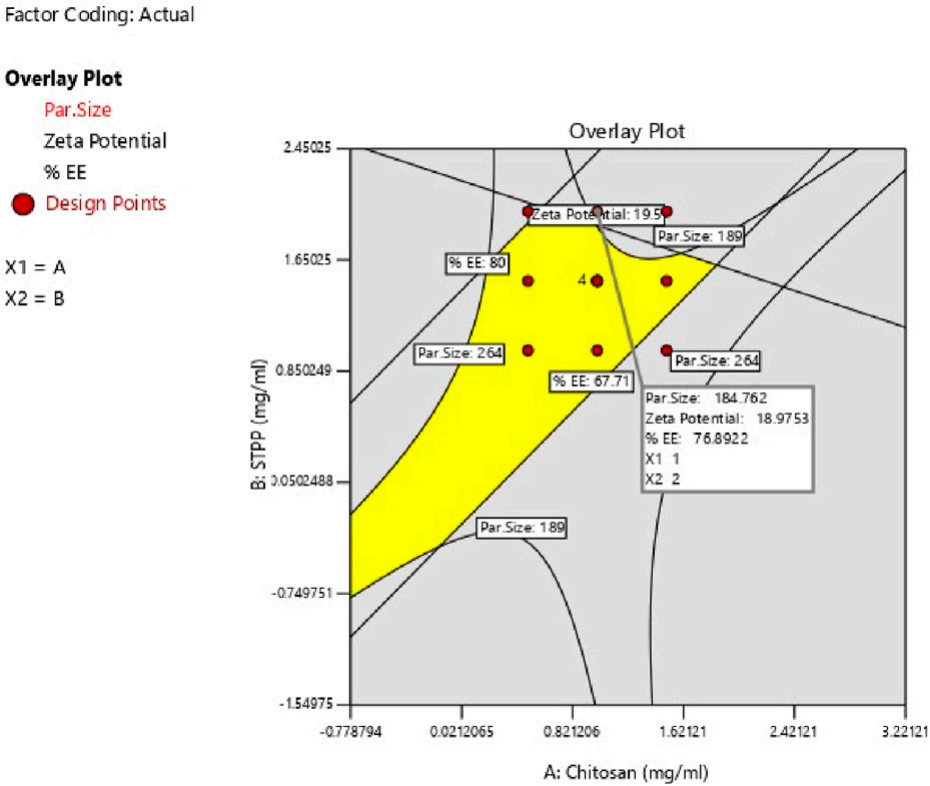
Overlay plot of Optimize batch.

The study aimed to identify the optimal combination of drugs and excipients to achieve the best formulation outcomes. A 3^2^ full factorial design consisting of 12 experimental runs was used to optimize the formulation. Desirability-based optimization was conducted to determine the optimal formulation parameters. Contour plots were produced for each response variable, and an overall overlay plot divided the area into desirable and undesirable zones. This methodical procedure guaranteed a comprehensive assessment of various determinants affecting nanoparticle properties, culminating in an optimized formulation. Because batch 8 performed better than the other formulations in terms of PS, PDI, ZP, and %EE, it was determined to be the optimal formulation. Batch 8 contained CS (1 mg/mL) and STPP (2 mg/mL) had acceptable PS of 189 ± 3.14 nm, ZP of 22.2 ± 1.25 mV, PDI of 0.372 ± 0.84 and %EE of 76.5% ± 1.64% and it fell in the yellow region. Figure 5 represents the yellow region as the optimized area.

### 3.5 Characterization of ESCI-loaded CSNPs

**FIGURE 6:**
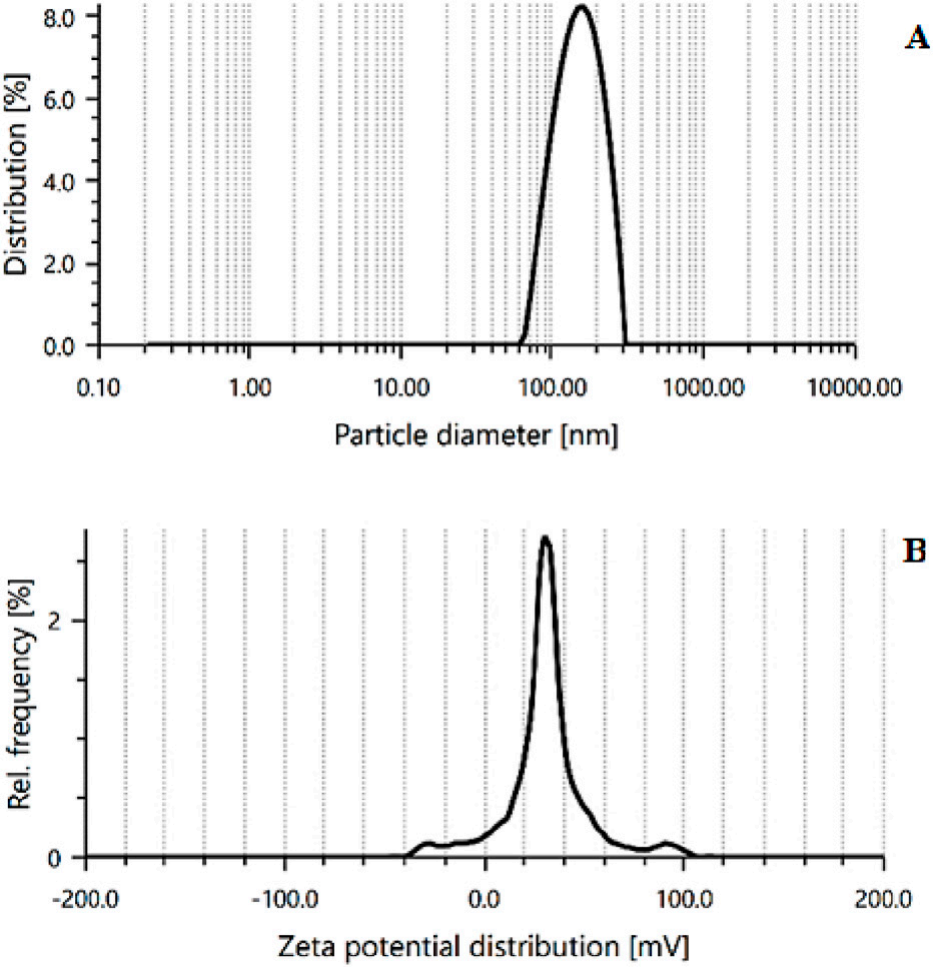
PS **(A)** and ZP **(B)** of optimized CSNPs (Batch 8).

The PS and PDI of optimized CSNPs were found 189 ± 3.14 nm, and 0.372 ± 0.84, respectively, as shown in Figure 6A. The PDI score shows good homogeneity and dispersibility of nanoparticles in the solution. ZP is a key indicator of particle stability, with a minimum of ±30 mV required for nanosuspensions stabilized solely by electrostatic repulsion to achieve physical stability (Shelke et al., 2016; Qi et al., 2021). The ESCI-loaded CSNPs demonstrated a mean ZP of +22.2 ± 1.25 mV (Figure 6B), reflecting adequate stability in the prepared formulation. This positive charge is the characteristic of CS/STPP nanoparticles and arises from the particle formation process. Negatively charged STPP molecules interact with positively charged amine groups on CS to neutralize certain charges, leaving residual amino groups to account for the positive ZP. The moderately high ZP value indicates that the ESCI-loaded CSNPs possess sufficient stability within the evaluated range (Kotta et al., 2022; Mujtaba et al., 2024b). The optimized formulation showed a DL of 21.89% ± 1.41% and a %EE of 76.5% ± 1.64%. These results suggested that ESCI could be efficiently entrapped in the CSNPs.

**FIGURE 7:**
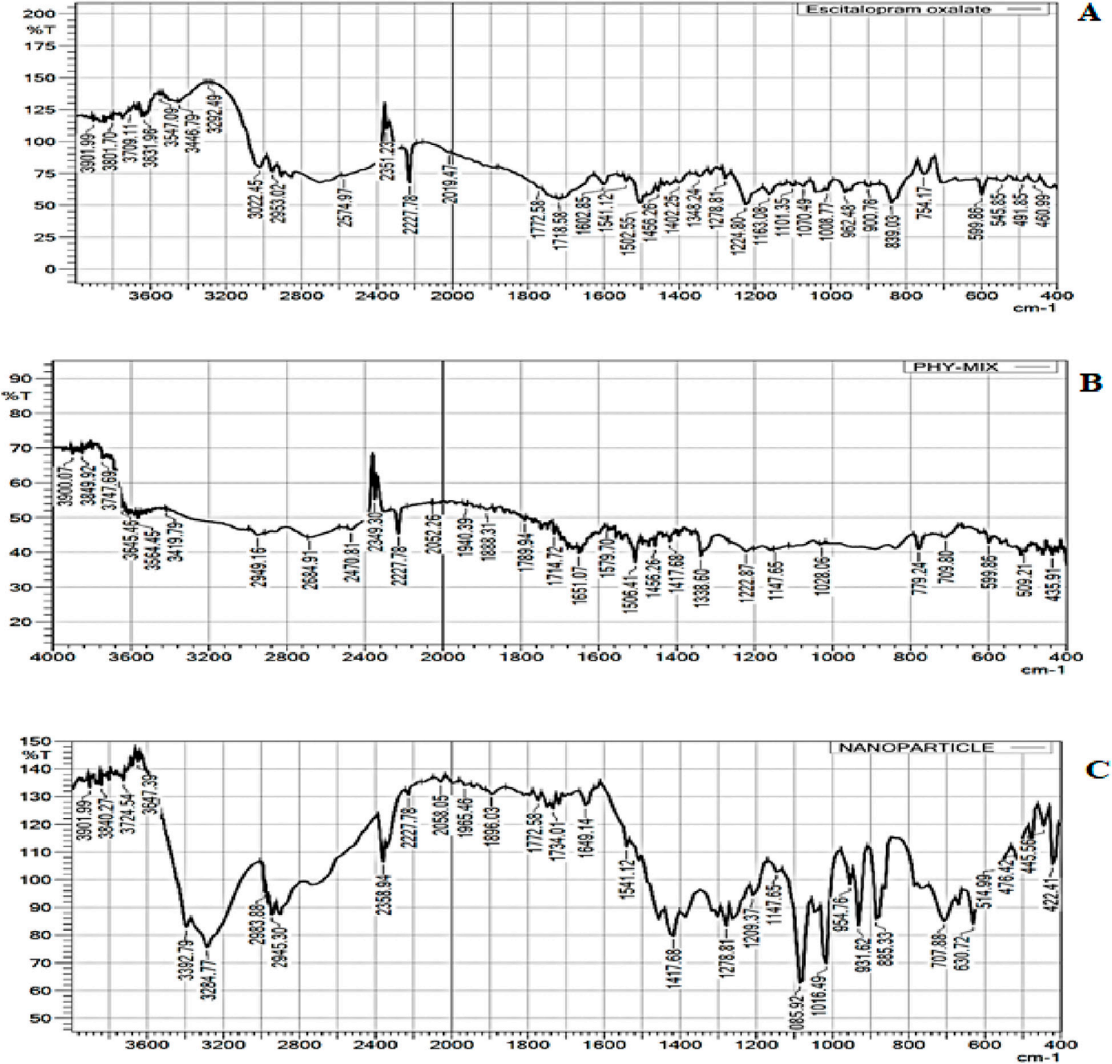
FTIR spectra of ESCI **(A)**, physical mixture **(B)**, and ESCI-loaded CSNPs **(C)**.

FTIR was performed to evaluate the intermolecular interactions in the formulations (Fule et al., 2023). FTIR spectroscopy shows the absorption bands of characteristics functional groups of ESCI at 2227.78, 1163.08, 1502.55, 1402.25, and 1070.49 cm^−1^ for C≡N stretching, -C-O stretching, C=C stretching, -CH_3_ stretching, C-C stretching respectively (Figure 7A). FTIR spectrum of ESCI revealed characteristic peaks representing the presence of functional groups claimed by its chemical structure. From this, it is considered that the drug is of pure quality. The physical mixture and ESCI-loaded CSNP formulation were found to retain the peaks that corresponded to the distinctive absorption bands and bonds of ESCI, following the interpretation of the FTIR spectrum (Figures 7B, C). Since there were no indications of drug-excipient incompatibility, it may be claimed that the drug remained in the formulation and physical mixture.

**FIGURE 8:**
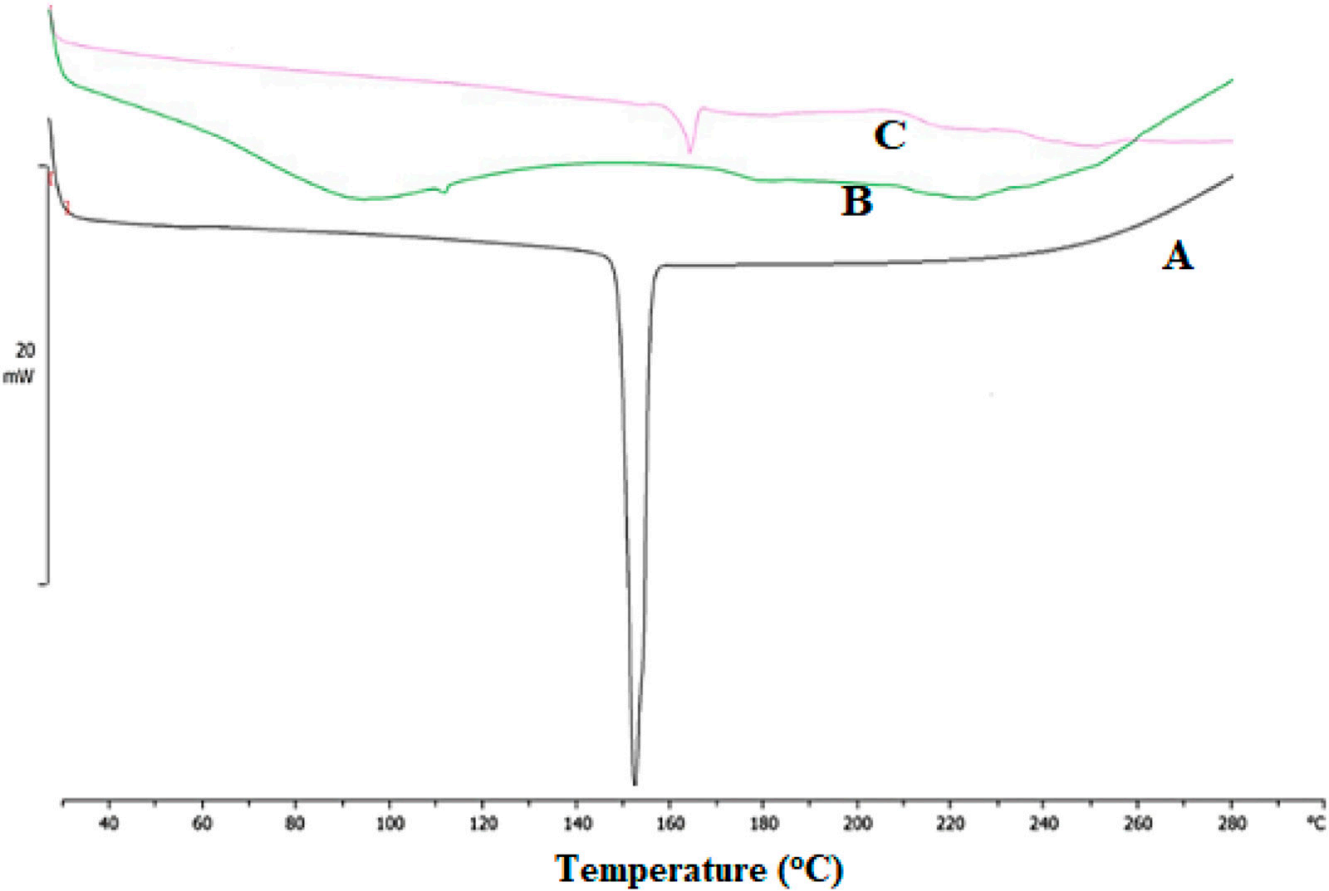
DSC thermogram of ESCI **(A)**, physical mixture **(B)**, and ESCI-loaded CSNPs **(C)**.

DSC is an effective tool for investigating the thermal behavior of a broad range of materials. Additionally, it offers data on the development of novel entities and interactions between drugs and excipients (Ali Mujtaba et al., 2024; Chiu and Prenner, 2011; Gill et al., 2010). DSC curves of ESCI, physical mixture, and CSNPs formulations were performed, and the results are revealed in Figure 8. The DSC thermogram of ESCI exhibited a sharp endothermic peak at 152.83°C which is the melting point of the drug indicating the existence of the drug in pure form and no degradation (Figure 8A). In the DSC data of formulated CSNPs, the endothermic peak was observed near to 165.35°C (Figure 8C). Melting endothermic peak of the drug was well observed with a slight change in broadening of peak or shifting towards higher temperature. The absence of this thermal transition in the ESCI-loaded CSNPs confirms the molecular dispersion in the formulations.

**FIGURE 9:**
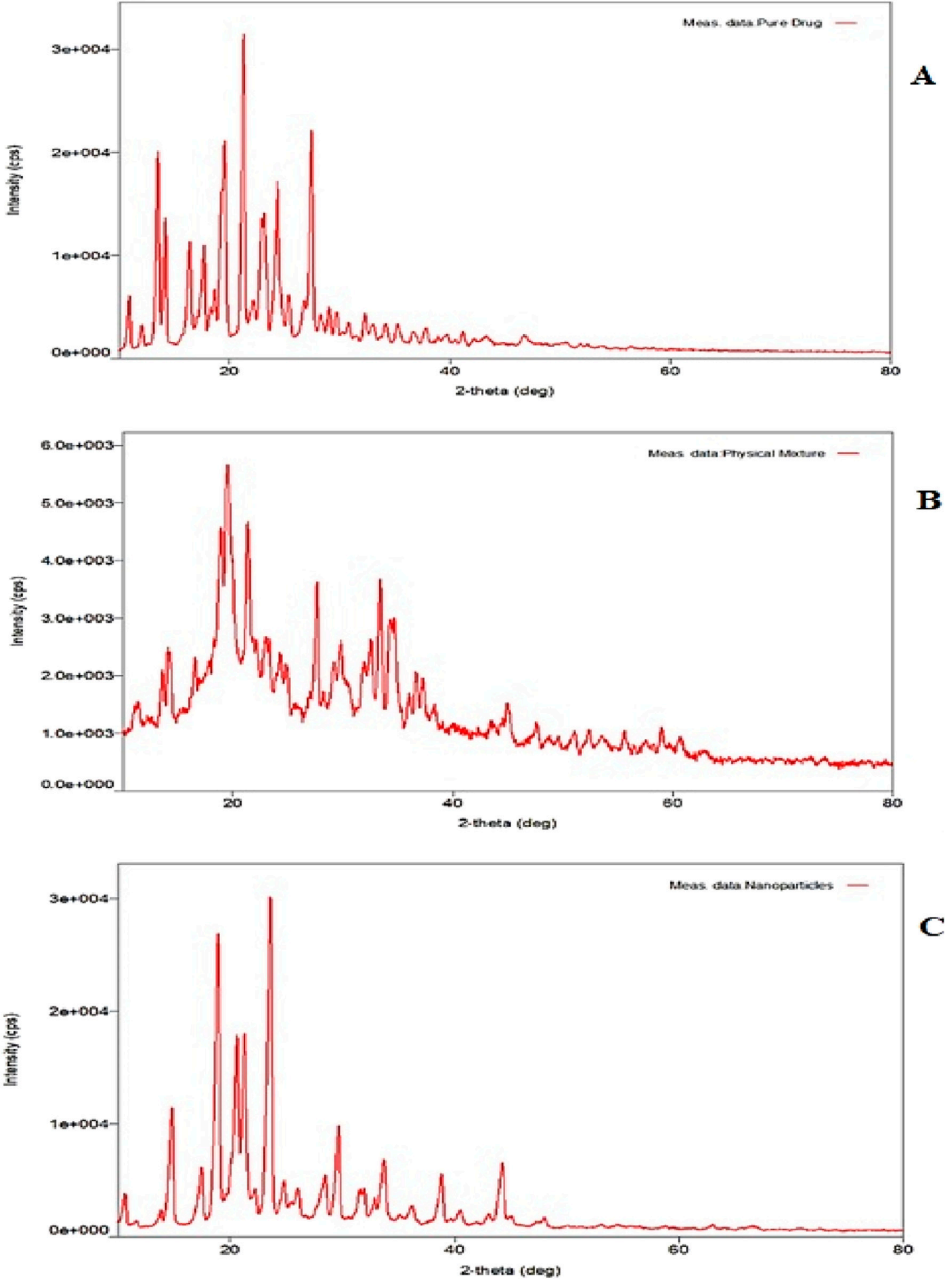
XRD pattern of ESCI **(A)**, physical mixture **(B)**, and optimized CSNPs **(C)**.

XRD is a powerful technique for identifying the polymorphs, changes in crystal habits, and the formation of new crystals during the preparation of CSNPs. The presence of sharp distinctive peaks at different angles in XRD confirmed the typical crystalline nature of the drug (Figure 9A). Figure 9B shows the XRD pattern of the physical mixture of drug and polymer. XRD pattern of ESCI-loaded CSNPs shows there is a decrease in the intensity indicating partial amorphization and entrapment of drug molecules (Figure 9C). Some sharp peaks present in the XRD pattern were due to the unentrapped molecule of the drug in the nanoparticles (Ali Mujtaba et al., 2024).

### 3.6 Formulation and assessment of CSNPs loaded *in situ* gel

Batch number 8 was selected as the optimized formulation and subsequently developed into an *in situ* gel for intra-nasal delivery to the brain. The nasal *in situ* gel of ESCI has been formulated using the cold method. The cold method involved the slow addition of Carbopol 940 (1.5%), HPMC (0.2%) polymers, lyophilized optimized CSNPs formulation (batch 8), and additives in cold water with continuous agitation. Carbopol 934 was used as a pH-sensitive or bio-adhesive polymer. The formed mixture was stored overnight at 4°C to get a clear gel. 1% benzalkonium chloride was used as a preservative to increase the shelf-life of the product. The solution was further characterized by various parameters. The prepared *in situ* gel observed a gelling time of 5.0 ± 0.41s, gelling pH of 6.26 ± 0.12, and viscosity of 44210 ± 116.43 cps. These systems are expected to undergo shear thinning (falling viscosity at increasing shear rate) due to the pseudoplastic behavior of the gel formed (Mohanty et al., 2023).

### 3.7 Analysis of drug release *in vitro* and release kinetics

**FIGURE 10:**
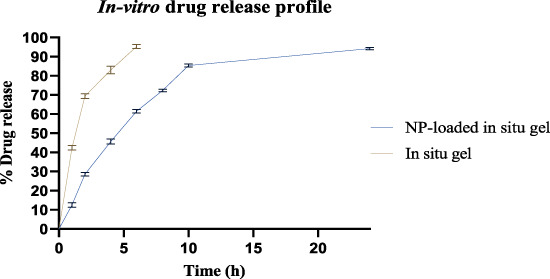
*In vitro* drug release of ESCI-loaded *in situ* gel and ESCI-loaded CSNPs *in situ* gel.

As illustrated in Figure 10, the dialysis-bag approach was used to assess the *in vitro* cumulative drug release patterns of both ESCI-loaded gel and ESCI-loaded CSNPs *in situ* gel. Within 4 h, the ESCI-loaded gel reached 96.3% ± 3.2, indicating a fast release. The ESCI-loaded CSNPs *in situ* gel, on the other hand, showed a biphasic release pattern and a continuous release, reaching 84.08% ± 2.24 over 10 h. During the first 4 hours, there was a burst release in the first phase, which was explained by either free ESCI molecules or those on the surface of the nanoparticles diffusing quickly. The controlled drug release mechanism of the CSNPs *in situ* gel was then demonstrated by a slower, continuous release over the next 24 h (Alam et al., 2012). Independent of the CSNPs, the *in situ* gel matrix acts as an extra barrier to control the release of ESCI. A more sustained and extended delivery profile is facilitated by the improved control over medication release offered by this dual-layered method. For a delivery strategy that accurately depicts the drug release or diffusion profile, it is necessary to study drug-release processes and kinetics. The drug release data of the ESCI-loaded CSNPs *in situ* gel formulation were fitted to numerous models, including the zero order, first order, Higuchi model, and Korsemeyer Peppas model. The kinetic model selected from the release study of ESCI-loaded CSNPs *in situ* gel followed predominantly the zero-order kinetic model, which means a constant rate of drug release with time, and it is independent of the concentration. Mathematical modeling indicated that the ESCI-loaded CSNP release followed the Higuchi model, with a high *R* ^2^ value of 0.9418 (Table 6), confirming that the drug was released primarily *via* a diffusion-controlled mechanism (Godbole et al., 2014).

**TABLE 6:** Release kinetic study of ESCI-loaded CSNPs *in situ* gel.

Models	Equation	*R* ^2^
Zero-order	y = 3.6403x + 19.912	0.7661
First-order	y = 0.0545x + 1.0434	0.4137
Hixson- Crowell	y = 0.1312x + 2.2178	0.4724
Korsmeyer-Peppas	y = 1.125x + 0.72	0.7144
Higuchi plot	y = 20.915x - 1.4738	0.9418

### 3.8 *Ex vivo* drug permeation study

**FIGURE 11:**
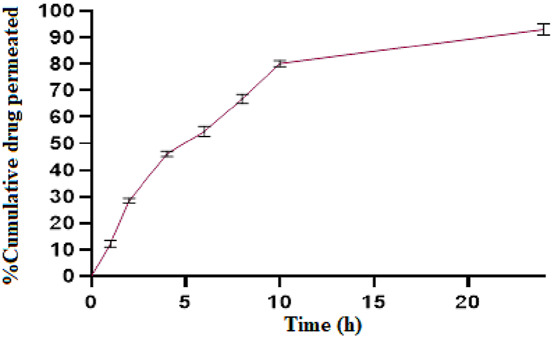
% cumulative drug permeation vs. time graph of ESCI-loaded CSNPs *in situ* gel through the nasal mucosa.

The permeation profile of ESCI-loaded CSNPs *in situ* gel through goat nasal mucosa is shown in Figure 11. The drug permeated after 8 h reached to 80.72% ± 3.12%. Using the Higuchi equation, the data was analyzed to validate the diffusion mechanism, yielding an *m* value of 2.866. This indicates that drug release occurs via a diffusion process regulated by Fick’s law, which is time-dependent and follows a square-root relationship. The drug release mechanism following the Higuchi model signifies that constant diffusivity and sink conditions are perfectly maintained in the release media (Mujtaba et al., 2024c).

### 3.9 Quantification of ESCI in biological samples and pharmacokinetic analysis

**FIGURE 12:**
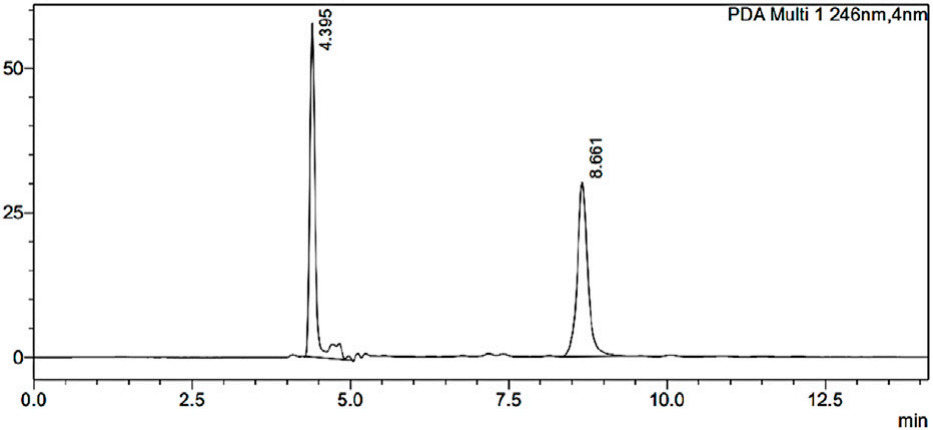
Optimized HPLC chromatogram of ESCI and internal standard in the presence of biomatrix at pH 7.6.

Reversed-phase HPLC (RP-HPLC) analysis was used to determine the ESCI concentrations in tissue and plasma samples from *in vivo* pharmacokinetic experiments after a liquid-liquid extraction process. The RP-HPLC method consists of a mobile phase comprising ammonium bicarbonate (25 mM) of pH 7.6 as mobile phase A, acetonitrile (ACN) as mobile phase B, its ratio of 85:50, with a flow rate of 0.5 mL/min, run time is 20 min, was found to be more suitable and well resolved and symmetrical peak. A 20 μL injection volume and a wavelength of 237 nm were used for detection. Figure 12 illustrates the separation of the peak of ESCI and Glecaprevir (IS) respectively without interference from plasma components. These specific optimized chromatographic conditions are shown in Table 7.

**TABLE 7:** Result of the estimated parameter in an optimized method of HPLC chromatogram.

Parameter	Escitalopram oxalate	Glecaprevir
Retention time (RT)	8.661	4.395
Peak area	346,665	358,178
Peak height	30,007	57,701
Tailing factor	1.099	1.266
Theoretical plates (USP)	13,792	11,220
HETP (USP)	10.875	13.368
Resolution factor (USP)	18.511	-
Capacity factor (k)	0.971	-

**FIGURE 13:**
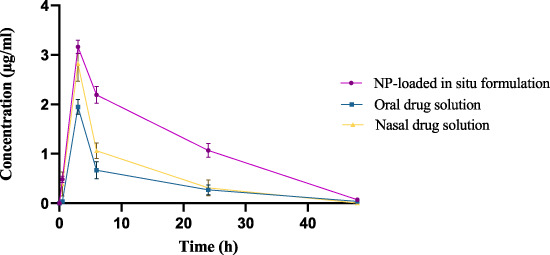
Drug concentration-time profile of CSNP *in situ* gel in plasma.

**FIGURE 14:**
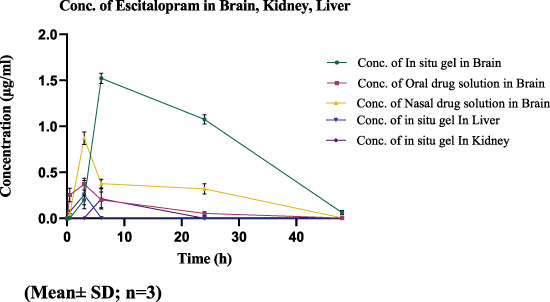
Drug concentration-time profile of CSNP *in situ* gel in Brain, Kidney, and Liver.

The drug concentration-time profile of ESCI-loaded CSNPs *in situ* gel in plasma, brain, kidney, and liver are shown in Figures 13, 14. The corresponding pharmacokinetic parameters in tissue and plasma obtained after i. n administration of the free ESCI, i. n administration of ESCI-loaded CSNPs *in situ* gel, and oral drug solution to rats are shown in Tables 8, 9. Following a single nasal dose of ESCI-loaded CSNPs *in situ* gel, the peak drug concentration (C_max_) in the brain reached 1.45 μg/mL. This concentration was significantly higher than that achieved with a standard intranasal drug solution (0.83 μg/mL) and a standard oral drug solution (0.31 μg/mL). The total AUC_0-12_ for the CSNPs *in situ* gel reached 38.87 μg.h/mL, significantly exceeding that of the standard intranasal drug solution (11.42 μg.h/mL) and the standard oral drug solution (2.92 μg.h/mL). The mean residence time (MRT) in the brain was also extended for the CSNPs *in situ* gel (17.43 h) as compared with the standard intranasal drug solution (14.43 h) and standard oral drug solution (9.86 h) reflecting an increased half-life. This higher C_max_ and prolonged MRT highlight the potential of CSNPs *in situ* gel as an effective brain-targeting system via the intranasal route. The T_max_ for both the standard intranasal drug solution and oral drug solution was 3 h, whereas the CSNPs *in situ* gel exhibited a delayed T_max_ of 6 h. This delay is likely due to the higher viscosity of the gel, which slows the release of NP from the formulation within the nasal cavity, resulting in more sustained drug release in the brain (Fazil et al., 2012). The rat’s sniffing movement before full gelation presumably removed some of the formulations from the nasal respiratory region, which contributed to the lower plasma C_max_ compared to the brain. This is why the C_max_ in plasma for CSNPs *in situ* gel was 0.40 μg/mL. These findings demonstrate that the *in situ* gel formulation delivers a higher concentration of ESCI to the brain compared to both oral and nasal drug solutions. Comparatively bioavailability assessments of intranasally administered ESCI solution and ESCI-loaded CSNPs *in situ* gel for brain targeting were conducted and statistically analysed. The results exhibited a substantial enhancement in bioavailability with the ESCI-loaded CSNPs *in situ* gel compared to the ESCI solution (*p* < 0.05), highlighting the efficacy of this formulation for direct nose-to-brain delivery in depression treatment. CS exhibits good mucoadhesive properties and has been demonstrated to enhance the paracellular transport of small molecules, biomolecules, and nanoparticles by modifying tight junction proteins. As a result, a significant amount of ESCI brain uptake was observed from CSNPs *in situ* gel formulation.

**TABLE 8:** Evaluation parameters of *In vivo* kinetic study in Tissue.

Parameters	i.n *In situ* formulation			Oral drug solution	i.n drug solution
	Brain	Liver	Kidney	Brain	Brain
T_max_(h)	6 ± 1	3 ± 0.5	6 ± 1	3 ± 0.2	3 ± 0.1
C_max_ (μg/mL)	1.45 ± 0.30	0.11 ± 0.01	0.10 ± 0.21	0.31 ± 0.23	0.83 ± 0.25
T_1/2_(h)	7.74 ± 1.20	5.8 ± 0.05	6.5 ± 0.62	8.36 ± 1.05	7.25 ± 0.12
MRT(h)	17.43 ± 3.21	4.7 ± 0.06	7.47 ± 0.31	9.86 ± 2.01	14.43 ± 0.54
AUC_0-12_ (μg.h/mL)	38.87 ± 1.02	0.5 ± 0.10	1.14 ± 0.21	2.92 ± 0.25	11.42 ± 3.02

**TABLE 9:** Evaluation parameters of *In vivo* kinetic study in Plasma.

Parameters	*i.n* *in situ* formulation	Oral drug solution	i.n drug solution
T_max_ (h)	3 ± 0.5	3 ± 0.2	3 ± 1
C_max_ (μg/mL)	0.40 ± 0.10	1.90 ± 0.62	2.52 ± 0.65
T_1/2_ (h)	12.2 ± 1.42	8.57 ± 0.14	4.20 ± 2.01
MRT(h)	7.17 ± 2.04	11.06 ± 2.05	7.48 ± 1.32
AUC_0-12_ (μg.h/mL)	1.78 ± 1.08	15.96 ± 0.54	19.76 ± 2.54

The plasma pharmacokinetic study, as revealed in Table 9 showed a significant difference in C_max_ where the oral drug solution exhibited the highest plasma concentration (1.90 ± 0.62 μg/mL) followed by i. n drug solution (2.52 ± 0.65 μg/mL), whereas the least C_max_ was observed in the i. n *in situ* gel formulation (0.40 ± 0.10 μg/mL). The comparison demonstrates that the CSNP-loaded *in situ* gel formulation provides a lower C_max_, indicating prolonged drug release and reduced incidence of systemic side effects because of increased intracellular delivery from nose to brain. Moreover, the T_1/2_ of the i. n *in situ* gel formulation (12.2 ± 1.42 h) was longer than the i. n drug solution (4.20 ± 2.01 h) and the oral drug solution (8.57 ± 0.14 h), suggesting prolonged retention of the drug. The AUC_0-12_ for the *in situ* gel formulation (1.78 ± 1.08 μg.h/mL) was lower than that of the i. n drug solution (19.76 ± 2.54 μg.h/mL) and oral drug solution (15.96 ± 0.54 μg.h/mL), which is consistent with the hypothesis that most of the drug that reaches the brain from the *in situ* gel formulation is not entering the systemic circulation. This supports the effectiveness of nose-to-brain drug delivery, which occurs through the action of CSNP and *in situ* gelation. The plasma and brain tissues pharmacokinetic results support the literature (S. M. More et al., 2025b). Thus, the current study supports that i. n delivery of ESCI-loaded CSNPs *in situ* gel enhances brain bioavailability and facilitates targeted brain delivery. These results validate its potential as an effective alternative to oral and conventional intranasal formulations for depression treatment.

## 4 Conclusion

In this study, we developed ESCI-loaded CSNPs *in situ* gel for intranasal delivery targeting the brain. CS and STPP (independent variables) were examined about PS, ZP, and %EE (dependent variables) using a 3^2^-factorial design. Optimization yielded a formulation with a PS suitable for brain delivery, a stable ZP, and a high %EE. ESC-NLCs were characterized using FTIR, DSC, and XRD analyses. The optimized CSNPs batch was incorporated into a pH-sensitive *in situ* gel matrix containing Carbopol 940 and HPMC K4M, prepared by cold method. The optimized nanoparticle-loaded nasal *in situ* gel-containing drug shows excellent gelation time, gelation pH, and suitable prolonged drug release. *In vivo* pharmacokinetic studies showed a higher brain concentration of ESCI in the *in situ* gel in comparison with standard treatment, underscoring the potential of this system as a non-invasive drug delivery approach for direct nose-to-brain targeting in depression. These findings suggest that drugs could reach the brain through the nose rather than the bloodstream. However, the research is in its early stages and needs substantial clinical data on appropriate higher animal models to assess its risk/benefit ratio and efficacy in humans.

## Data Availability

The original contributions presented in the study are included in the article/supplementary material, further inquiries can be directed to the corresponding authors.
